# Medical education during the Covid-19 pandemic long-term experiences of German clinical medical students

**DOI:** 10.1371/journal.pone.0286642

**Published:** 2023-06-06

**Authors:** Marc Gottschalk, Pascal M. Milch, Christian Albert, Katrin Werwick, Ruediger C. Braun-Dullaeus, Philipp Stieger

**Affiliations:** 1 University Clinic for Cardiology and Angiology, Center for Internal Medicine, University Medicine Magdeburg, Magdeburg, Germany; 2 Clinic for Nephrology, Central Clinic Bad Berka, Bad Berka, Germany; 3 Deans Office of Student Affairs, Medical Faculty, Magdeburg University, Magdeburg, Germany; American University of Beirut Medical Center, LEBANON

## Abstract

**Introduction:**

Due to the Covid-19 pandemic and the accompanying hygiene regulations, medical students in Germany faced multiple educational and personal challenges. The challenges included the cancellation and digitalisation of courses, the closing of university institutions such as libraries, a decrease in social contacts, and the risk of a Covid-19 infection. The aim of this study was to understand medical students’ pandemic experiences as well as the consequences of these experiences for the students’ future work as physicians.

**Materials and methods:**

We performed 15 guided, one-on-one interviews with clinical medical students (third to fifth year) at the Otto-von-Guericke-University Magdeburg. Interviews were recorded, transcribed, and anonymised. We performed a qualitative content analysis in accordance with Mayring and thereby formed an inductive category system. The Consolidated Criteria for Reporting Qualitative Research (COREQ) were applied.

**Results:**

Five categories were inductively formed: “Changes in the teaching experience”, “negative effects on the learning experience”, “decrease in personal social contacts”, “contact with covid-19”, and “pandemic-associated stress increase”. The participating students reported higher levels of stress due to isolation and uncertainty regarding their educational future. Furthermore, students welcomed the digitalisation of lectures, developed individual coping strategies, and voluntarily took part in the care of Covid-19 patients. Limitations to social interactions were perceived as the major restrictive factor to their educational structure, their perceived learning success and personal development.

**Conclusion:**

This study identified social restrictions as well as didactic and academic structural challenges as relevant factors contributing to perceived stress and fear for medical students during the Covid-19 pandemic, especially as regards their learning experience. Students’ acceptance of digitalised learning may enable regular interaction with university peers and may facilitate a structured educational life. However, the implementation of digital resources could not provide a sufficient substitute for in-person courses.

## Introduction

Due to the Covid-19 pandemic, German medical students experienced major changes in their education starting in February 2020. Contact restrictions and other hygiene regulations had impact on clinical education, and affected the socialisation and learning experiences of many students [1, 2]. In March 2020, nationwide contact restrictions were established [3], which resulted in the suspension of in-person courses and the temporary closing of community institutions such as libraries, restaurants, and clubs. At the same time, there was a lack of resources to control Covid-19: The health care system was running low on personal protection equipment [4, 5], point of care antigen tests were unavailable until the fall of 2020 [6, 7] and vaccinations did not start until January 2021 [8].

Medical faculties, therefore, chose to convert many classes to digital teaching with facing a different extent of preparation for students and lecturers [9, 10]. We recently reported on the perceived lack of preparedness regarding the example of a medical school such as Magdeburg medical faculty, where technical and didactic challenges and the absence of social contacts were recognised as limitations of academic distance teaching, too [11]. From the students’ perspective, academic peers fulfil an important role for individual learning success and medical socialisation [12, 11]. Emerging studies suggest [13, 14], that pandemic restrictions confounded this social arrangement, eventually leading to subpar learning performance. Considering these findings [13, 14] it still remains unclear, how the aforementioned pandemic regulations and local restrictions affected medical students’ long-term learning experience.

Accordingly, using qualitative content analysis of guided interviews, this study aimed to investigate on students’ experience of academic medical education at the Otto-von-Guericke University Hospital Magdeburg during the Covid-19 pandemic and the potential implications of these experiences for their future work as physicians. We hypothesised, that the combination of social distancing and curricular digitalisation influenced the students’ perceived learning success.

## Materials and methods

Based on the intent to understand and improve upon the learning experience of medical students, we performed a qualitative interview study with 15 clinical medical students (third to fifth year) of the Otto-von-Guericke-University Magdeburg after 15 months of medical education under pandemic regulations. Data were collected from April to July 2021. We used guided one-on-one interviews in combination with an established and standardised method of qualitative content analysis according to Mayring [15, 16]. The Consolidated Criteria for Reporting Qualitative Research (COREQ) is an established tool used to improve data reporting concerning reflexivity, study design, and data analysis in qualitative research [17]. Therefore, the proposed methodology was applied, and the corresponding protocol is provided as S1 Table. The study was approved by the institutional review board of the Otto-von-Guericke-University, Medical Faculty Ethics Committee, Magdeburg, Germany (Case No. 49/21). All data were obtained after detailed participant information. Consent to participate was obtained and documented.

The recruitment of participants was accomplished by making use of direct contacting and a modified snowball-system [18] in order to establish a maximal diversity concerning gender, age, origin, and residential situation. The sample (Table 1) consisted of 15 clinical students with an average age of 25 years (Median 24, SD 3,44), 60% of the participants were female. Most students were living alone (47%) or in a shared flat (27%), a further 20% were living with their spouses. Also, most students (80%) had moved to Magdeburg from another city. The average interview length was 52 minutes (min. 33 –max. 73 minutes).

**Table 1:** Characteristics of the sample used in the qualitative study.

Characteristic	Expression
**Sex, % (n)**	40% (6) male
	60% (9) female
**Mean age**	25,33 years (Median 24; SD 3,44)
**Type of housing, % (n)**	26,7% (4) living in a shared flat
	46,7% (7) living alone
	20% (3) living with partner
	6,7% (1) living with parents
**Place of origin, % (n)**	20% (3) born in Magdeburg
	73,3% (11) born in Germany, moved to Magdeburg
	6,7% (1) born abroad, moved to Magdeburg
**Residence during the pandemic, % (n)**	93,3% (14) stayed in Magdeburg
	6,7% (1) did not stay in Magdeburg

**Fig 1:**
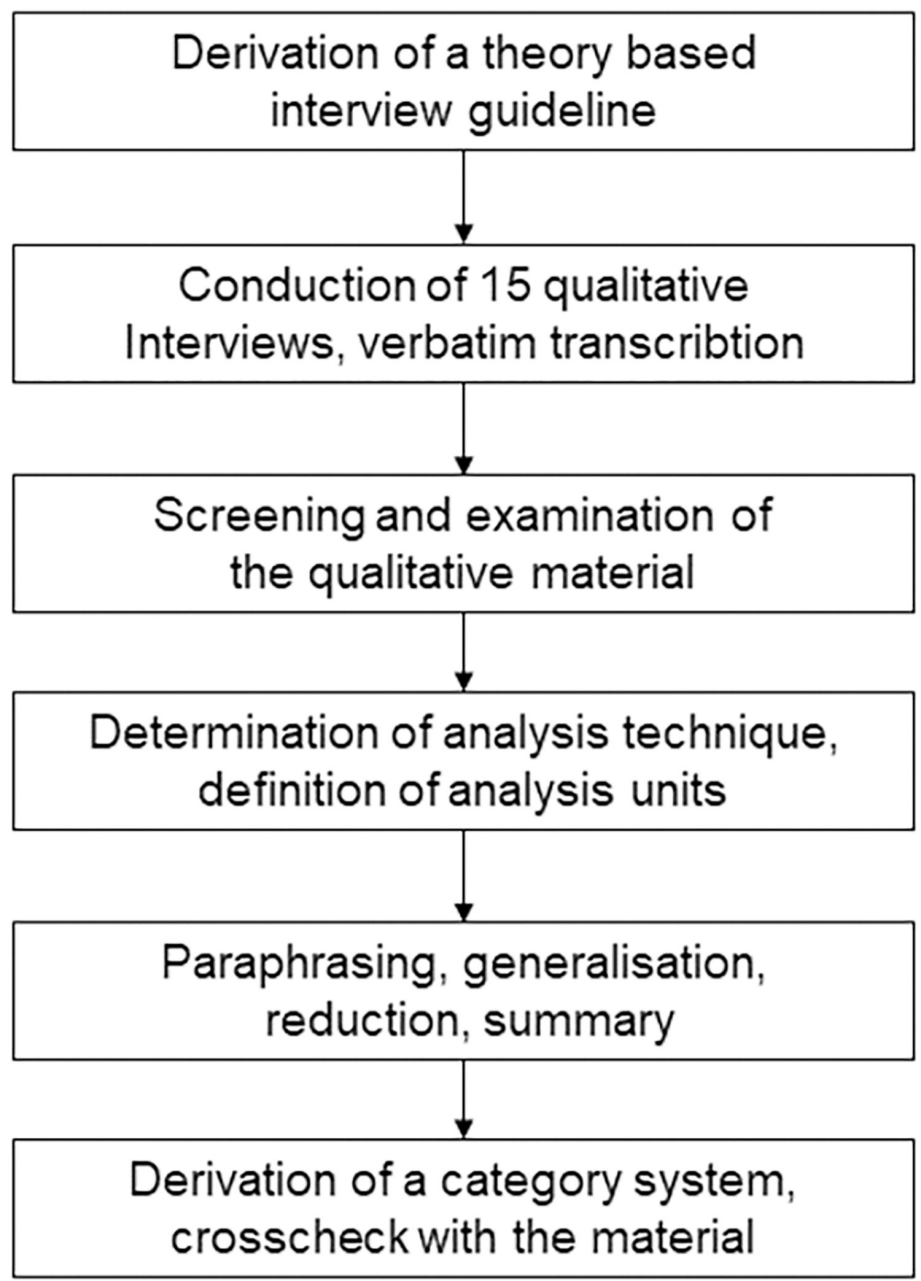
Study flowchart.

The interview guide was created in November 2020 after performing literature research regarding dynamics in medical education as well as findings associated with the Covid-19 pandemic. In November 2020 for this literature research MEDLINE (through PubMed interface, http://www.ncbi.nlm.nih.gov/pubmed) and Web of Science, specifically as proposed by Meinefeld, were used. [19]. The literature-based guide (S2 Table) consisted of 30 questions, which addressed the following themes: study experiences during the Covid-19 pandemic, social influences of the Covid-19 pandemic, or effects of the pandemic on becoming a physician. The questionnaire was piloted on two medical students, and subsequently adapted by PM and MG. Due to pandemic restrictions, all interviews were performed via phone calls by PM. The interviews were carried out in German. On the selected sample material, we used forward and backward translation to ensure semantically appropriate English translations of the content. Field notes were obtained concerning the interview atmosphere and demographics. The interviews were recorded, transcribed, and anonymised [20]. To identify citations each interview was given an anonymised code. A qualitative content analysis was performed using Microsoft Excel (Version 1808, Redmont, Washington, USA). After sighting and reflecting of the material, the analysis units were defined and a sequence of generalisation, reduction, and summation was performed. An inductive category system was thus formed, and, this was later validated on the remaining material [15]. To optimize transparency and reproducibility, coding was performed by two separate researchers (MG, PM) [16]. A communicative validation of the inductively formed categories, followed by a saturation discussion, was performed by MG and PM using a video conferencing system [21]. Participant validation was not performed. The study flowchart is shown in Fig 1.

## Results

We inductively identified five main categories concerning the students’ perceived educational experience during the Covid-19 pandemic: “Changes in the teaching experience”, “negative effects on the learning experience”, “reduction of personal social contacts”, “contact with Covid-19”, as well as “pandemic associated stress increase”. These consisted of 17 subcategories that are summarized in Table 2.

**Table 2:** Inductively formed categories and subcategories.

Category	Subcategory
**Changes in the teaching experience**	Opportunities and advantages of digital courses
	Didactic challenges
	Organisational challenges
	Lack of clinical experience
**Negative effects on the learning experience**	Reduction of contact with academic peers
	Loss of structure
**Reduction of personal social contacts**	Influence of contact restrictions
	Prioritising of real-life contacts
	Inner conflicts in managing contacts
	Adjustment of leisure time activities
**Contact with Covid-19**	Moderate fear of Covid-19
	Fear of transmitting Covid-19 to others
	Being quarantined
	Covid-19 infections and contact situations
	Care for Covid-19 patients
**Pandemic associated stress increase**	Pandemic associated stress symptoms
	Coping strategies for pandemic associated stress

The first category “changes in the teaching experience” contained four subcategories: “Opportunities and advantages of digital courses”, “didactic challenges”, “organisational challenges” and “lack of clinical experience”. “Opportunities and advantages of digital courses” described the potential of digitalised classes. Students experienced more flexibility with respect to the time and learning location. The acceptance of online lectures was generally high. Students reported higher participation rates than during real life lectures. Many students shared the opinion that online and especially on-demand courses may be a good addition to the existing curriculum. However, there were many “didactic challenges”: Not all courses were easily amenable to digital teaching, especially seminars and practical exercises were reported to be challenging. While seminars could be optimised to establish a sufficient degree of interactivity, practical activities often could not be taught adequately according to the students. For example, students explicitly preferred in-person clerkships:

*„And I would just wish, […] that the internships were held in person, at least in the skills lab and in small groups, too.” (I5.4_39, lines 333–335)*

Also, insufficiently prepared lecturers and technical barriers were perceived as significant challenges. Furthermore, we identified several “organisational challenges”: Lecturers lacked technical and didactical training, communication between students and the university was experienced as slow and sometimes ineffective, and practical courses, including clerkships, would be suspended at short notice depending upon the changing pandemic regulations.

In the second category “negative effects on the learning experience” two major problems were described: The first was a considerable “reduction of contact with academic peers”. Due to contact restrictions students were not able to meet with other students and discuss their studies and topics associated with their medical education. This resulted in a feeling of isolation, demotivation, and a perceived less efficient exam preparation. For example, the participant in interview I5.1 reported missing recreational breaks with other students:

*“Taking breaks together with your mates, to come down, relax or have lunch together, you need this between courses, to be able to concentrate again. These are rather trivial things, but they are somehow missing” (I5.1_42, lines 335–338)*

Students tried to compensate the lack of social interaction by making use of messengers and video-conference tools, but the results were perceived as insufficient:

*“[…] sometimes there are even discussions during the lecture. With [name of video-conferencing software], there are one or two who really still do that. But, in general, I would say that most find it more pleasant to be anonymous, turn the monitor off, say nothing, and withdraw, […]” (I3.5_10, lines 86–89)*

The second major problem could be described as “a loss of structure”. The pandemic regulations led to a reduction in real life activities. Also, essential (learning) locations like the library or the cafeteria were shut down, forcing the students to stay at home facing suboptimal conditions:

*“[…] as a result of not having this structure and this control over when you do what, you also often stray. And then at the end of the day, you realize how ineffective you were and that really stresses me out.” (I4.1_53, lines 482–484)*

Students encountered limited access to learning resources as well as noise and distraction form roommates and neighbours. To counter this loss of academic structure, students started to use self-made timetables, oriented their daily routine around meals or activities, and started part-time student jobs.

The category “reduction of personal social contacts” refers to the effects of pandemic regulations on the students’ social life. In terms of the “influence of contact restrictions”, the students had difficulties in maintaining existing contacts and establishing new ones, especially during times with strict pandemic regulations:

*“I haven’t had any contact with some people since the beginning of the pandemic. Especially with people from the university, […]" (I3.4_47, lines 611–617)*

In addition, students reported, that they started “prioritising of real-life contacts”. Students only met with a few close friends or family members, respecting the contact restrictions on the one hand and preserving at least a small number of social contacts on the other:

*“And perhaps that one has also made a bit of a cutback. As silly as that sounds. That you just say: Okay, with whom would I definitely like to meet now? And with whom does it not necessarily have to be now?” (I5.2_35, lines 369–371)*

Furthermore, we could identify the subcategory “inner conflicts in managing contacts”: Students experienced stress and uneasiness when weighting contact restrictions and infection risk against the wish to meet with friends. This is exemplified by the students’ perspective in Interview I4.1:

*“And if you were someone who already paid a lot of attention to it, then you were just completely out of it and you weren’t asked anymore. I actually witnessed that once or twice in my private life, so it was an extreme social break, I have to say.”(I4.1_45, lines 394–399)*

To cope with contact restrictions, students made an “adjustment of leisure time activities”. Due to the impossibility of going out or taking part in group activities, students reported to invest more time into sports and outdoor activities or going for walks. Furthermore, there was an increase in phone calls and video conferences with friends or relatives:

*“For example, I signed up for an online sports course and do it once a week or sketch or something. So things that you can do well at home alone, so that you don’t just always have the same routine every day." (I5.1_49, lines 395–399)*

In the fourth category “contact with Covid-19” we summarized the student’s experiences and perceptions concerning the virus. It became apparent that students had a “moderate fear of Covid-19”. Most medical students were not concerned about requiring hospitalisation or intensive care treatment if they were to be infected because they perceived themselves to be rather young and healthy. In contrast, Long Covid was regarded as a comparably greater threat. Furthermore, the students felt a relevant “fear of transmitting Covid-19 to others”. Students were highly sensible of harming patients or family members, who potentially had a higher risk of severe courses with Covid-19. The participant in interview I5.5 for example described his fears:

*“[…] Or that I infect someone else, for example. And that other person will somehow bring it home to his grandparents, and they will then die from it. That’s present, of course, even now. It worries me.” (I5.5_44, lines 260–262)*

Another problem that became especially apparent before medical licensing exams was the fear of “being quarantined” and thus unable to take the exam. In the case of formal medical licensing examinations, this could lead to waiting times of up to half a year. In addition, students in our sample experienced “Covid-19 infections and contact situations” themselves. No severe courses were reported in our sample, but students described difficulties in the handling of contact information of persons at risk and access to Covid-19 testing. Remarkably, despite their fears and the risk of being quarantined after accidental contact to Covid-19-infected person, a fair share of our participants took part in the “care for Covid-19 patients” by working in test-centres, fever clinics or on Covid-19 wards.

The fifth category, “pandemic associated stress increase” is closely connected to the previously described categories and may be regarded as a consequence of multiple factors like social isolation and perceived diminished productivity. In the subcategory “pandemic associated stress symptoms” students described sleeping disorders, a feeling of unrest, and, in some cases, psychosomatic symptoms. However “coping strategies for pandemic associated stress” could also be identified. Students tried to do things with which they had positive associations. They communicated with family and friends using the phone or computer, and they tried to establish new hobbies, focussing especially on outdoor activities as described for example in the interview I5.4:

*“But otherwise, I just do more sports now, I think, more cycling, running. Then I go for a walk with friends. I now also have contact in the evening via video calls, somehow also online games with friends or here, friends, family.“(I5.4_53, lines 460–463)*

**Fig 2:**
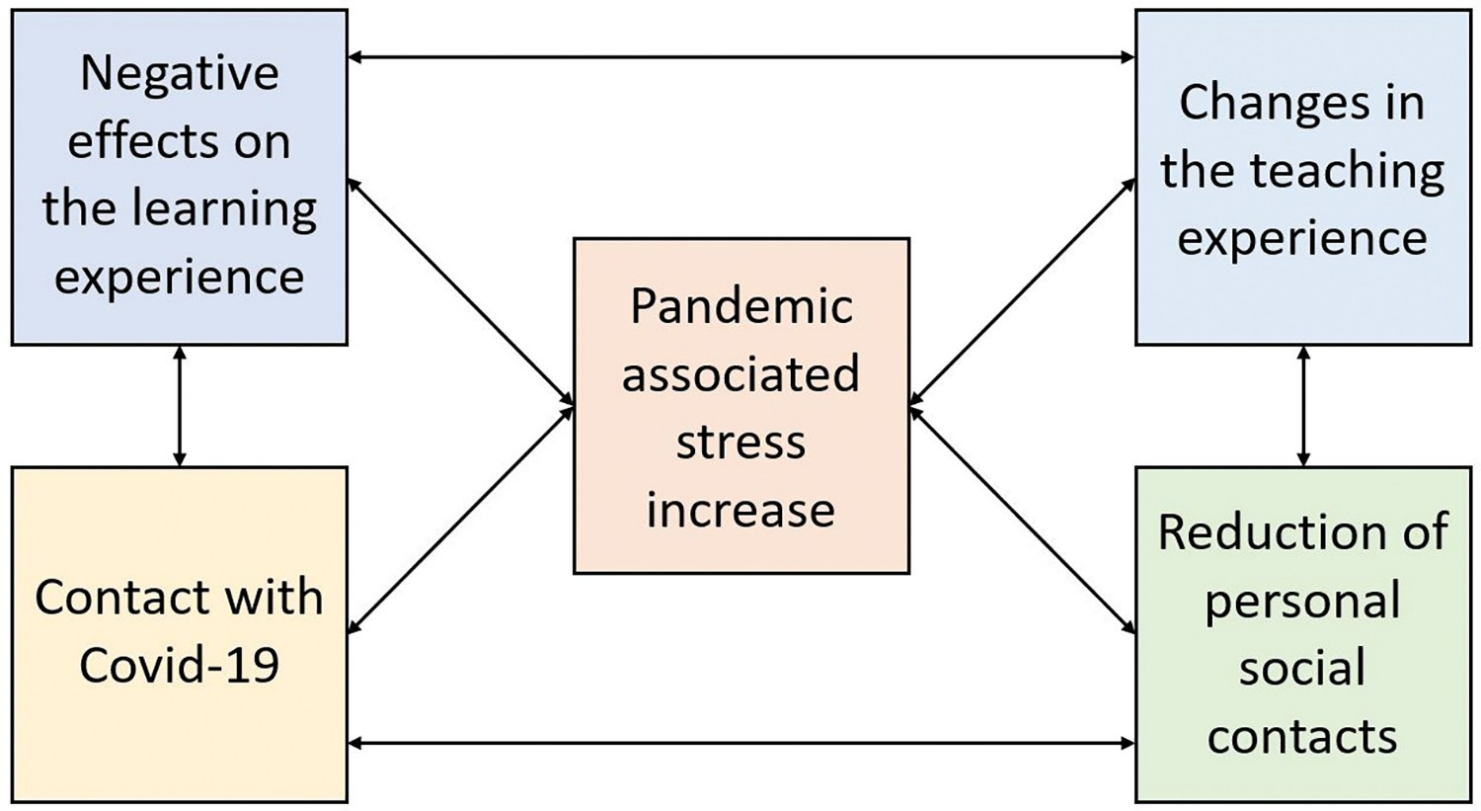
Category model: An illustration of the category model derived from the interview material.

The results are illustrated in a category model (Fig 2), which highlights the interdependencies of the categories derived from the interview material. The “pandemic associated stress increase” may be seen as a central motive associated with the experienced changes in the learning and teaching experiences and in the students’ social arrangements.

## Discussion

We performed guided one-on-one interviews with clinical medical students at the Otto-von-Guericke-University Magdeburg University to evaluate their perceived experiences of academic medical education during the Covid-19 pandemic. Analysing this example of coping with pandemic challenges of an university faculty, we inductively identified five main categories: “Changes in the teaching experience” “negative effects on the learning experience”, “reduction of personal social contacts”, “contact with Covid-19”, as well as “pandemic associated stress increase”. The participating students reported higher stress levels that were caused by social isolation and uncertainty regarding their educational future. The importance of maintaining peer and professional relationships was emphasised, as social relationships fulfil an important role for students to an extent that cannot be achieved with online teaching.

On the other hand, students welcomed the digitalisation of lectures, developed individual coping strategies, and took part voluntarily in the care for Covid-19 patients. All changes in educational procedures and social routine were meaningful, and the students had considerable concerns about personal and general aspects regarding the future.

Concerning the teaching experiences during the Covid-19 pandemic, multiple studies have described lectures, seminars, and practical courses that were made available via online learning platforms and via online conference systems [22–26]. For example, AlQhtani et al. [27] and Zheng et al. [28] described few limitations in digitalized practical training. In contrast, our participants experienced these formats as suboptimal, as likewise described by Djermester et al. [23]. Furthermore, teachers form several universities have reported technical challenges associated with hard- and software solutions [11, 29, 30]. Although many courses were digitalised and initially well received, didactical hurdles arose secondary to the limitations of distance teaching [11] as well as from local deficiencies in faculty preparedness [31]. According to Di Giacomo [33], another important factor is the lack of a structured online curriculum, which was locally reported by the participants of our study. However, the interplay and impact of social, institutional, and teaching restrictions on students’ perceived learning success were not assessed in detail in German cohorts of medical students during the COVID-19 pandemic [29, 32, 33].

In the present study, students reported a perceived decrease in clinical experience and preparedness due to restrictions on practical education in clerkships, skills labs, and wards. Consequently, this increased their worries regarding potential negative effects on their exam performances. Building upon findings from our prior study, which evaluated the learning experiences of Magdeburg students in the medical clerkship prior to Covid-19 [34], social exchanges with peers were again found to be important. The students in our present study were severely influenced by the lack of contact with their peers. Similar findings were observed by Di Giacomo and Di Paolo for dentistry students [35]. Specifically, in the present study social interaction, relationships, and contacts were found to play an important role in the students’ life and in the learning environment to an extent that cannot be replicated with online teaching alone. Others have suggested that more frequent supervision by mentors and a diverse array of meeting platforms may be helpful to improve the progression in biomedical and medical programs [36].

Additionally, the negative effects of contact restrictions on social interaction of students in the present study are in line with studies concerning adults [37] and adolescents [38] in Germany. The need for certain social restrictions, specifically in the educational sector, continues to be debated retrospectively, and the potential negative effects of such restrictions on society and economy is being increasingly recognised [39–41]. Such findings are also in accordance with the present material.

The theme of voluntarily taking care for Covid-19 patients, which emerged in the material, was previously described by Mühlbauer et al. [42], who identified the wish to help and a sense for duty as the most important reasons. Nikendei et al. [43] reported on the benefits of taking part in the care of Covid-19 patients in terms of personal growth and identification with the profession, which could be partly found in our material. The reported lack of fear of becoming infected and experiencing a severe course is in line with findings from the United States [44], which reported a comparably lower perceived risk in young adults with academic education. Furthermore, Ciancio et al. [44], stated that young academics perceived a higher risk of infection and transmission than non-academics or older people, leading to a better adherence to hygiene measures, which was reflected in our material on numerous occasions. The fear of being excluded from medical licensing exams due to quarantine, which was reported by our study participants, is shared in the medical community: Hammoud et al. [45] expressed their concerns regarding the USMLE examinations and consequently suggested the introduction of more flexible licensing rules.

Qualitative research offers a broad approach that leaves room for the experiences and perspectives of a sample of individuals, which thus enables the formation of new hypotheses and models [15, 46]. A major limitation is the small number of interviews conducted, and this may restrict the generalisation of our research results. This is however typical for qualitative approaches as discussed by Steinke [16]. Limitations might also arise from our recruiting method, potentially introducing selection-bias. However, this methodology is established [18] and enabled straightforward accessibility of a student cohort and successful performance of the study considering COVID-19 restrictions. Another limitation might arise from the single-centre sampling. However, most of the themes of the present results are in line with the international literature [23, 30, 35], which at least in part suggests the generalisability of our findings. Furthermore, we focussed on the students’ experiences, leaving aside factors such as individual characteristics and skills, although confounding may be possible.

Our study highlights the importance of social contact with university peers and the need for structure to the daily life of medical students. When adapting curricula to fit Covid-19 restrictions, measures should be taken to ensure an ongoing exchange between students and their peers [47]. Furthermore, universities should provide time management concepts, particularly when in-person studies are reduced to a minimum [48]. Still, digitalised learning formats have high potential for future curricular development. Especially regarding schedule optimisation [49]. Improvements in digitalisation, teaching, and adaptation of medical curricular specifically for the structured integration of practical and theoretical courses may be needed in order to support students, who are completing their medical training during unusual circumstances, such as the Covid-19 pandemic [50].

## Conclusion

This study identified social restrictions and didactic or structural academic challenges as relevant factors leading to perceived stress for medical students during the COVID-19 pandemic, especially as these factors relate to their present and future learning experiences. Our findings highlight the importance of interaction with university peers and the need for both a structured educational curriculum and daily life to cope with pandemic restrictions. Students were open to digitalised courses for lectures, but limitations emerged for practical courses, which may have led to deficiencies in practical and communicational skills. The implementation of digital resources could not provide a sufficient substitute for in-person training.

## Supporting information

S1 Table COREQ checklist. (PDF)

S2 Table Interview guideline. (DOCX)

S3 Table Categories, subcategories and anchor quotations. (DOCX)
