# Supplementary material for: Medical education during the Covid-19 pandemic long-term experiences of German clinical medical students
Source: PLoS One. 2023 Jun 6;18(6):e0286642. doi: 10.1371/journal.pone.0286642 (PMC10243622; doi:10.1371/journal.pone.0286642)
Supplement: S2 Table — (DOCX) [file pone.0286642.s002.docx]

**S2 Table Interview Guideline**

| **Question No.** | **Question (German)** | **Question (English translation)** |
| --- | --- | --- |
| 1 | Worin lagen für sie Vorteile der digitalen Lehre? | What do you think were the advantages of digital teaching? |
| 2 | Worin lagen für Sie Nachteile der digitalen Lehre? | What were the disadvantages of digital teaching? |
| 3 | In welchem Umfang haben Sie die angebotenen Online-Vorlesungen genutzt? | To what extent have you used the online lectures provided by your faculty? |
| 4 | Worin lagen für Sie die Stärken und Schwächen der Vorlesungen? | What do you think were the strengths and weaknesses of the lectures? |
| 5 | Wie zufrieden waren Sie mit den online Seminaren? | How satisfied were you with the online seminars? |
| 6 | Welche Umsetzungen der Seminare waren besonders effektiv? | Specifically, which implementations of the seminars did you find effective? |
| 7 | Welche Umsetzung von Praktika während der Pandemie empfanden Sie als erfolgreich? | Which implementation of internships during the pandemic did you consider successful? |
| 8 | Welche Kriterien stellen Sie dabei an ein erfolgreiches Praktikum? | What criteria do you set for an internship in order to consider it successful? |
| 9 | Wie schätzen Sie die Qualität der Lehre im Vergleich zu einem regulären Semester mit Präsenzunterricht ein? Bitte begründen Sie Ihre Aussage. | How do you rate the quality of online teaching courses compared to those of regular semesters with face-to-face classes? Please give reasons for your assessment. |
| 10 | Was hat sich für Sie an der Prüfungsvorbereitung geändert? | How did exam preparation change for you? |
| 11 | Wie hat sich die Durchführung der Prüfungen verändert und wie sind Sie damit umgegangen? | How has the conduct of the exams changed and how did you deal with it? |
| 12 | Welche Unterschiede zwischen dem Sommersemester 2020 und dem darauffolgenden Wintersemester 2020/21 haben Sie in der Lehre feststellen können? | What differences have you noticed in teaching of curricular courses regarding the summer semester in 2020 and the winter semester of 2020/2021? |
| 13 | Welche Änderungen würden sie für ein weiteres Onlinesemester vorschlagen? | What changes would you suggest for another online semester? |
| 14 | Würden Sie Teile der digitalen Lehrinhalte für den regulären Lehrbetrieb beibehalten wollen und wenn ja, welche und warum? | Would you like to retain parts of the digital teaching content for regular teaching and if so, which ones and why? |
| 15 | Haben Sie während der Pandemiezeit eine oder mehrere Famulaturen absolviert? | Did you complete one or more clinical clerkships during the pandemic period? |
| 16 | Wie hat/haben sich diese von bisherigen Famulaturen unterschieden? | How was/were this/they different from previous clinical clerkships? |
| 17 | Wie haben Sie das Sozialleben auf dem Campus in Zeiten der Pandemie wahrgenommen? | How did you perceive social life on campus during the pandemic? |
| 18 | Welche Aspekte des Campuslebens vermissen Sie? | What aspects of campus life do you miss? |
| 19 | Wie hat sich die Pandemie auf Ihre sozialen Kontakte im Allgemeinen ausgewirkt? | How has the pandemic affected your social contacts in general? |
| 20 | Welche Auswirkungen gab es speziell auf die Kontakte zu Ihren Kommilitonen? | What impact did it have specifically regarding interaction with your fellow students? |
| 21 | Haben sie während der Pandemiezeit Symptome von Stress oder Depression wahrgenommen? | Did you percieve symptoms of stress or depression during the pandemic? |
| 22 | Haben Sie für sich Bewältigungsstrategien entwickeln können? | Have you been able to develop coping strategies for yourself? |
| 23 | Haben Sie während der Pandemiezeit eine Nebentätigkeit aufgenommen oder eine bereits bestehende Tätigkeit ausgeübt, falls ja berichten Sie davon. | During the pandemic, did you take up a second job or engage in an existing one, if so please elaborate. |
| 24 | Wie haben sie die Rolle der Universität bezüglich des Informationsmanagements während der Pandemie empfunden? | How did you feel about the university’s role regarding information management during the pandemic? |
| 25 | Hatten Sie das Gefühl jederzeit seitens der Universität über aktuelle Regelungen etc. aufgeklärt zu sein? | Did you feel that you were always informed by the university about current regulations, etc.? |
| 26 | Welchen Einfluss haben die Lehrbedingungen auf den Austausch mit Ärzten? | What impact do teaching conditions have on interactions with physicians? |
| 27 | Welchen Einfluss haben die Lehrbedingungen auf Ihre medizinischen Erfahrungen in der Praxis gehabt? | What influence did the teaching conditions have on your practival medical experience? |
| 28 | Was denken Sie, welchen Einfluss wird die Pandemie auf Ihre spätere Tätigkeit als Arzt/Ärztin haben? | What impact do you think the pandemic will have on your future work as a physician? |
| 29 | Bestehen bei Ihnen Sorgen oder Ängste durch die Pandemiebedingungen eine schlechtere Ausbildung zu erhalten? Bitte begründen sie ihre Einschätzung! | Do you have any concerns or fears that you will receive less medical education due to the pandemic conditions? Please elaborate! |
| 30 | Wie hat sich die Pandemie auf ihre praktische Erfahrung mit Patienten, Ärzten und dem Gesundheitswesen ausgewirkt? | How has the pandemic affected your hands-on experience with patients, physicians, and the health care system? |
