# Supplementary material for: Medical education during the Covid-19 pandemic long-term experiences of German clinical medical students
Source: PLoS One. 2023 Jun 6;18(6):e0286642. doi: 10.1371/journal.pone.0286642 (PMC10243622; doi:10.1371/journal.pone.0286642)
Supplement: S3 Table — (DOCX) [file pone.0286642.s003.docx]

**Supplemental Table 3: Categories, subcategories, and anchor quotations**

| **Category** | **Subcategory** | **Anchor Quotation (Translated from German)** | **Anchor Quotation (German)** |
| --- | --- | --- | --- |
| **Changes in the teaching experience** | Opportunities and advantages of digital courses | *„[...] if there are three of you in a breakout session, then you cannot skip reading the text or something like that. And you also can’t have only two people discussing it, and you stay out of it. I thought that was definitely very good.“(I4.3_12, lines 121-123)*  *„[...] I find it quite good because you can watch them when you want. I also find it an advantage for example that you can now listen to lectures again from time to time.“ (I4.2_5, lines 50-54)* | *„[...] wenn man zu dritt in einer Breakout-Session ist, da kannst du nicht den Text nicht gelesen haben oder so. Und es geht auch nicht, dass nur zwei Leute diskutieren, und du hältst dich raus. Das fand ich auf jeden Fall sehr gut.“ (I4.3_12, Z. 121-123)*  *„[...]Das finde ich ganz gut, weil, die kann man sich dann anschauen, wann man möchte. Das zum Beispiel finde ich jetzt auch einen Vorteil, dass man Vorlesungen sich immer wieder einmal anhören kann,.“ (I4.2_5, Z. 50-54)* |
|  | Didactic challenges | *„[...] the seminars per se were simply far too long. I think you can’t sit concentrated in front of the PC for three or four hours. If something then again does not work somehow, then that is simply just junk.“ (I4.1_12, lines 108-113)*  *„I would say that one of the weaknesses was that perhaps no questions could be asked. They would need to be formulated separately as an email, and whether every lecturer can answer every student, as one would simply do in the lecture hall, is, of course, questionable. [...].“ (I5.2_9, lines 98-103)* | *„[...] die Seminare an sich waren einfach viel zu lange. Ich finde, man kann nicht drei, vier Stunden vor dem PC konzentriert sitzen, und wenn dann irgendwie wieder was nicht funktioniert oder irgendwie was nicht klappt oder so, das ist einfach richtig Käse.“ (I4.1_12, Z.108-113)*  *„Die Schwächen, würde ich sagen waren, dass vielleicht keine Fragen gestellt werden konnten, die hätte man separat als E-Mail formulieren müssen. Und ob dann auch wieder jeder Dozent jedem Studenten so antworten kann, als würde man es einfach im Vorlesungssaal machen, ist natürlich fragwürdig. [...].“ (I5.2_9, Z. 98-103)* |
|  | Organisational challenges | *„Well, in the beginning, nothing here really worked at all. No information was forthcoming because some people didn't know anything themselves. [...] Everything came too late, and there were problems everywhere with the technology here and there. Anyway, now it has definitely become much better..“ (I3.4_37, lines 468-471)*  *„Yes. I would say difficult in that a lot of information somehow then came I don’t know how, pieces of it also contradictory and a bit late.“ (I4.4_52, lines 412-413)* | *„Also am Anfang hat hier wirklich gar nichts funktioniert, da kamen keine Infos, weil man teilweise selber nichts wusste [...] es kam alles zu spät und überall gab es Probleme mit der Technik und hier und da. Also jetzt ist das auf jeden Fall viel besser geworden.“ (I3.4_37, Z. 468-471)*  *„Ja. Ich würde sagen schwierig also, dass da viele Infos irgendwie, ich weiß nicht, zum Teil auch widersprüchlich und ein bisschen spät dann gekommen sind.“ (I4.4_52, Z.412-413)* |
|  | Lack of clinical experience | *„[...] And we will clearly start the practical year with deficits. [...] from a purely practical point of view, I don’t think that it is easy to compensate for that, and we’ll just have to make up for a lot things in the practical year..“ (I5.2_48, lines491-499)*  *„But especially the things in the clinic, they could not be completely replaced. There were admittedly good block practicums. In anesthesia we now had one day in the skills lab, there you did learn a lot. […]However, that should have been a week where you're also in the operating room and so see much more." (I5.4_11, lines 75-79)* | *„[...] Und wir werden mit Defiziten ganz klar ins PJ starten. [...] rein praktisch gesehen, so glaube ich nicht, dass man das so easy kompensieren kann und dass wir dann Vieles einfach im PJ nachholen müssen.“ (I5.2_48, Z.491-499)*  *„Aber gerade halt die Sachen in der Klinik, die konnten halt nicht ganz ersetzt werden. Es gab schon gute Blockpraktika. Also, Anästhesie hatten wir jetzt einen Tag im Skills Lab, da hat man schon viel gelernt. […] ansonsten wäre das eine Woche gewesen, wo man auch im OP ist und das alles, also, viel mehr sieht.“ (I5.4_11, Z. 75-79)* |
| **Negative effects on the learning experience** | Reduction of contact with academic peers | *„I would say that one of the weaknesses was that perhaps no questions could be asked. They would need to be formulated separately as an email, and whether every lecturer can answer every student, as one would simply do in the lecture hall, is, of course, questionable. [...].“ (I5.2_9, lines 98-103)*  *„Common breaks, this balance that you need between the courses to be able to concentrate again afterwards, or to be able to go out to eat together, even if these are quite banal things, but somehow that is missing.“ (I5.1_42, lines 335-338)* | *„Die Schwächen, würde ich sagen waren, dass vielleicht keine Fragen gestellt werden konnten, die hätte man separat als E-Mail formulieren müssen. Und ob dann auch wieder jeder Dozent jedem Studenten so antworten kann, als würde man es einfach im Vorlesungssaal machen, ist natürlich fragwürdig. [...].“ (I5.2_9, Z. 98-103)*  *„Gemeinsame Pausen, dieser Ausgleich, den man braucht zwischen den Lehrveranstaltungen, um sich danach wieder konzentrieren zu können, oder gemeinsam essen gehen zu können, auch wenn das ziemlich banale Sachen sind, aber irgendwie fehlt das schon.“ (I5.1_42, Z. 335-338)* |
|  | Loss of structure | *„Because one simply has a bit more,- yes, as a result of not having this structure and this control over when you do what, you also often stray. And then at the end of the day, you realize how ineffective you were and that really stresses me out.* *So when I realize: Okay, now I somehow didn't manage to do what I wanted to do, which actually wouldn't have been a problem, that stresses me out more than it did before.“ (I4.1_53, lines 481-486)*  *„That you somehow always sit in the same room. That you basically always see the same thing, that you don't have any variety. And that afterwards, for example, you somehow don't have any sense of time anymore.“ (I3.3_2, lines 22-24)* | *„Weil man einfach ein bisschen mehr-, ja dadurch, dass man nicht so diese Struktur hat und nicht so diese Kontrolle darüber, wann man was tut, schweift man halt auch oft ab. Und dann am Ende des Tages merkt man, wie ineffektiv man war und das stresst mich extrem. Also wenn ich merke: Okay, ich habe jetzt irgendwie gar nicht das geschafft was ich schaffen wollte, was eigentlich kein Problem gewesen wäre, das stresst mich schon mehr als es das vorher gemacht hat.“* *(I4.1_53, Z. 481-486)*  *„Dass man immer in dem gleichen Raum sitzt irgendwie. Dass man im Prinzip immer das Gleiche sieht, dass man keine Abwechslung hat. Und dass man nachher irgendwie auch keine Zeiteinschätzung zum Beispiel mehr hat.“ (I3.3_2, Z. 22-24)* |
| **Reduction of personal social contacts** | Influence of contact restrictions | *„That is somehow a bit of a double-edged sword. Well, some contacts have been completely shelved, so to speak. I haven't had any contact with some people since the beginning of the pandemic. Especially with people from the university,.[...]“ (I3.4_47, lines611-617)*  *"But in the examination phase, when you really should be sitting in front of your desk from morning to night, and then you don't even have to go somewhere and briefly see other people [...]". (I4.1_25, lines 211-213)* | *„Das ist irgendwie so ein bisschen so ein zweischneidiges Schwert. Also manche Kontakte halt so quasi völlig ad acta gelegt. Also mit manchen Leuten hatte ich gefühlt seit Beginn der Pandemie überhaupt keinen Kontakt mehr. Gerade so mit Leuten aus der Uni,.[...]“ (I3.4_47, Z.611-617)*  *„Aber in der Prüfungsphase, wenn man irgendwie wirklich von morgens bis abends vor dem Schreibtisch sitzen sollte, und dann noch nicht mal den Weg hat irgendwo hinzufahren und kurz mal andere Leute zu sehen […]“ (I4.1_25, Z. 211-213)* |
|  | Prioritising of real-life contacts | *„And perhaps that one has also made a bit of a cutback. As silly as that sounds. That you just say: Okay, with whom would I definitely like to meet now? And with whom does it not necessarily have to be now?“ (I5.2_35, lines369-371)*  *„And then one really paid even more extreme attention to with whom you were meeting. So it was really just two or three people, and the rest you didn't see at all.“ (I5.3_2 lines 26-27)* | *„Und vielleicht, dass man auch ein bisschen so Abstriche gemacht hat. So doof das klingt. Dass man halt sagt: Okay, mit wem möchte ich mich jetzt auf jeden Fall treffen? Und mit wem muss es jetzt nicht unbedingt sein?“ (I5.2_35, Z.369-371)*  *„Und da hat man ja wirklich noch viel krasser darauf geachtet, mit wem man sich trifft. Also das waren halt wirklich so zwei, drei Leute, und den Rest hat man gar nicht mehr gesehen.“ (I5.3_2 Z. 26-27)* |
|  | Inner conflicts in managing contacts | *„And if you were someone who already paid a lot of attention to it, then you were just completely out of it and you weren't asked anymore. I actually witnessed that once or twice in my private life, so it was an extreme social break, I have to say.“ (I4.1_45, lines394-399)*  *„And it also puts me in situations where I'm uncomfortable, where I think: Wow, there's quite a crowd of people here now." (I4.1_57 lines 523-524)* | *„Und wenn man halt jemand davon war der schon dolle darauf geachtet hat, dann war man halt einfach komplett raus und man wurde auch nicht mehr gefragt. Das habe ich im privaten Bereich tatsächlich ein, zweimal mitbekommen, also es war schon ein krasser sozialer Einschnitt muss ich sagen.“ (I4.1_45, Z.394-399)*  *„Und es bringt mich auch in Situationen, wo ich mich unwohl fühle, wo ich so denke: Wow, hier sind jetzt aber doch ganz schön viele Leute auf einem Haufen.“ (I4.1_57 Z. 523-524)* |
|  | Adjustment of leisure time activities | *„Somehow I also found it important now in the pandemic, when you can't do so much in groups, that you don't completely disregard yourself and your own hobbies. For example, I signed up for an online sports course and do it once a week or sketch or something. So things that you can do well at home alone, so that you don't just always have the same routine every day.” (I5.1_49, lines 394-399)*  *„"Yes, so, it was like, sometimes I thought it would be really nice to go out and, yes, party or sit in the pub one night and do something different for once." (I4.2_40, lines 345-347)* | *Irgendwie fand ich es auch wichtig jetzt in der Pandemie, wo man nicht so viel in Gruppen machen kann, dass man sich und die eigenen Hobbys nicht so ganz außer Acht lässt. Ich habe mir zum Beispiel einen Online-Sportkurs eingetragen und mach den einmal die Woche oder zeichnen oder so. Also Sachen, die man gut allein zu Hause machen kann, sodass man nicht einfach immer den gleichen Tag hat jeden Tag. (I5.1_49, Z. 394-399)*  *„Ja, also, es war so, dass ich manchmal gedacht habe, es wäre richtig schön, einmal irgendwie hinauszugehen und, ja, feiern zu gehen oder einmal abends in der Kneipe zu sitzen und einmal irgendetwas anderes zu machen.“ (I4.2_40, Z. 345-347)* |
| **Contact with Covid-19** | Moderate fear of Covid-19 | *„Well, I'm not afraid of dying from Corona, but I'm worried that if I do get Corona, that I could get the long-term damage. Yeah, that’s actually the only thing." (I5.5_43 lines 258-260)*  *„Yes, sure definitely afraid, notwith respect to ending up somehow in the hospital or intensive care unit, but rather just from this Long Covid." (I3.1_24, lines 304-305)* | *„Also ich habe keine Angst, an Corona zu sterben. Aber ich habe Sorge, dass wenn ich Corona bekommen könnte, dass ich die Langzeitschäden davontragen könnte. Das ist so, ja, eigentlich das einzige.” (I5.5_43 Z. 258-260)*  *„Ja klar auf jeden Fall Angst insofern, nicht mit irgendwie im Krankenhaus oder Intensivstation zu landen, aber halt vor diesem Long Covid“ (I3.1_24, Z. 304-305)* |
|  | Fear of transmitting Covid-19 to others | *„Or that I infect someone else, for example. And that other person will somehow bring it home to their grandparents,and they will then die from it. That's present, of course, even now. It worries me." (I5.5_44, lines 260-262)*  *„For example, Christmas 2020, when everything was still very uncertain and the incidences were high, I have to say that partly before Christmas, or let's say in the time before Christmas, I made sure that I didn't see anyone if possible, except for one friend, but of other friends actually none, because I wanted to travel to my family. It was more important for me not to see anyone and to be sure that I couldn’t be infected with Corona than to have contacts.", (I5.1_44, lines 350-356)* | *„Oder, dass ich jemand anderes anstecke, zum Beispiel. Und dieser andere bringt das irgendwie nach Hause zu den Großeltern, und sie werden dann daran sterben. Das ist natürlich präsent, auch jetzt. Macht mir Sorgen.“ (I5.5_44,Z.260-262)*  *„Zum Beispiel Weihnachten 2020, als das alles noch sehr unsicher war und die Inzidenzen hoch waren, da muss ich sagen habe ich teilweise vor Weihnachten, oder sagen wir in der Zeit vor Weihnachten, habe ich darauf geachtet, dass ich möglichst gar keinen sehe, bis auf einen Freund, aber so an Freunden eigentlich gar keinen, weil ich da zu meiner Familie fahren wollte und da war mir das wichtiger da niemanden zu sehen und sicher sein zu können, dass ich eigentlich kein Corona haben kann, mit dem ich mich infiziert habe, als dass ich da Kontakte habe.“ (I5.1_44, Z. 350-356)* |
|  | Being quarantined | *„So I was also actually infected, that's why I also had to – personally, I also had to, spend two weeks in quarantine here in Magdeburg. (I3.1_24, lines 302-303)*  *„So I don't know, I also think it's still extreme with the preliminary medical examination, that, in the groups, a lot of people really didn’t do anything with anyone or somehow in panic mode tried to have as little contact as possible, so as to not just somehow end up in quarantine. As then, the preliminary examination at the end of the year is postponed.“*  *(I3.2_1 lines 40-42)* | *„Also ich war auch infiziert tatsächlich, deswegen musste ich auch-, persönlich musste ich auch zwei Wochen in Quarantäne hier in Magdeburg.“ (I3.1_24, Z. 302-303)*  *„Also ich weiß nicht, finde ich auch noch krass mit Physikum, dass in den Gruppen wirklich viele Leute mit gar keiner was gemacht oder irgendwie panisch versucht hat, so wenig Kontakt wie möglich, dass man bloß nicht irgendwie in Quarantäne rutscht. Weil dieses Physikum dann am Ende des Jahres sich verschiebt.“*  *(I3.2_1 40-42)* |
|  | Covid-19 infections and contact situations | *„If you have symptoms or contacts, however, it is more difficult. Then you are only allowed to go to the outpatient fever clinic if you have a certain number of symptoms. General practicioners didn't test me at all.* *It was relatively difficult to then get a test." (I3.5_43, lines 417-420)*  *„[…], My aunt supposedly also had a positive test result two weeks ago, and we all had to go into quarantine. But it was just nothing, everybody was negative, even though we all had contact with her." (I3.4_58 lines 703-707)* | *„Wenn man aber dann Symptome hat oder Kontaktpersonen, ist es schwieriger. Dann darf man aber auch nur in die Fieberambulanz, wenn man eine gewisse Anzahl von Symptomen hat. Hausärzte haben mich gar nicht getestet. Es war dann relativ schwierig, dann an einen Test zu kommen.“ (I3.5_43, Z. 417-420)*  *„[…], meine Tante hatte auch angeblich vor zwei Wochen ein positives Testergebnis und da mussten wir halt alle in Quarantäne. Aber es war halt nichts, also es waren auch alle negativ, obwohl wir alle mit ihr Kontakt hatten.“ (I3.4_58 703-707)* |
|  | Care for Covid-19 patients | *„I also actually worked a bit on a Covid ward during my clinical elective, which means I actually also saw people who died from Covid. I particularly didn’t like the circumstances because they always died alone.“ (I4.2_45, lines 381-384)*  *„I mean, I used to work in the outpatient fever clinic, too, and, indeed, a lot of us still do.“ […]“ (I4.5_1 lines 208-216)* | *„Ich habe auch auf einer Covid-Station jetzt während der Famulatur tatsächlich ein bisschen gearbeitet, das heißt, ich habe tatsächlich auch Menschen gesehen, die an Covid gestorben sind. Ich fand die Situation aber auch nicht gut, weil die immer alleine gestorben sind.“ (I4.2_45, Z. 381-384)*  *„Ich meine, ich habe auch mal in der Fieberambulanz gearbeitet, und viele von uns machen das ja immer noch […]“ (I4.5_1 208-216)* |
| **Pandemic associated stress increase** | Pandemic associated stress symptoms | *“So, had therapy before, related to other things, very little of the other therapy. OK. These seminars and Zoom meetings especially, have caused me stress, yes, and also this home-alone learning and creating pressure for myself is, as I said, not for me. It is difficult for me. And yes, finding social contacts and this constant routine is not so good.“ (I3.5_36 lines 374-381)*  *„No, I had more the feeling that you were unproductive. And then you built up this kind of internal stress." (I5.3_53 lines 475-476)* | *“Also auch, hatte auch vorher Therapie, das hat sich noch auf andere Sachen bezogen, sehr wenig die andere Therapie-. Okay. Ja, vor allem diese Seminare und Zoom-Meetings haben bei mir Stress ausgelöst und auch dieses Zuhause-alleine-Lernen und sich selbst Druck schaffen ist, wie gesagt, nicht meins. Das ist auch schwierig für mich. Und ja, nicht so gut, in sozialen Kontakten finden und auch dann diese ständige Übung.“ (I3.5_36 Z. 374-381)*  *„Ne, ich hatte eher, dass man das Gefühl hat, man ist unproduktiv. Und dann hat man so einen inneren Stress aufgebaut.“ (I5.3_53 Z. 475-476)* |
|  | Coping strategies for pandemic associated stress | *„[...]And now I'm also working here part-time. That's the thing that gives me structure, working twice a week, then once a week a face-to-face event. [...]“ (I3.1_22, lines 261-268)*  *„But otherwise, I just do more sports now, I think, more cycling, running. Then I go for a walk with friends. I now also have contact in the evening via video calls, somehow also online games with friends or here, friends, family.“ (I5.4_53, lines 460-463)* | *„[...] Und jetzt arbeite ich hier auch noch nebenjobmäßig. Das ist so die Sache die mir Struktur gibt, zweimal die Woche arbeiten, dann einmal die Woche eine Präsenzveranstaltung. [...]“ (I3.1_22, Z. 261-268)*  *„Aber ansonsten, ich mache halt mehr Sport, glaube ich, jetzt, mehr Fahrrad fahren, Laufen mache ich. Dann geht man mal spazieren mit Freunden. Ich habe halt jetzt auch abends Kontakt über so Videoanrufe, irgendwie auch online was spielen mit Freunden oder hier, Freunde, Familie.“ (I5.4_53, Z. 460-463)* |
